# Manual Small Incision Cataract Surgery in Phacomorphic Glaucoma: Surgical Technique and Outcome in North-eastern India

**DOI:** 10.5005/jp-journals-10008-1136

**Published:** 2013-05-09

**Authors:** Vidyarani Rajkumari, Khongbantabam Singh Kaminibabu, Rajkumari Devi Bhabanisana, Rajkumar Victor

**Affiliations:** Assistant Professor, Department of Ophthalmology, Jawaharlal Nehru Institute of Medical Sciences, Imphal, Manipur, India; Senior Consultant and State Program Officer, Department of Ophthalmology, Jawaharlal Nehru Institute of Medical Sciences, Imphal, Manipur, India; Professor and Head, Department of Ophthalmology, Jawaharlal Nehru Institute of Medical Sciences, Imphal, Manipur, India; Senior Resident, Department of Ophthalmology, Jawaharlal Nehru Institute of Medical Sciences, Imphal, Manipur, India

**Keywords:** Phacomorphic glaucoma, Intraocular pressure, Intraocular lens implantation, Manual small incision cataract surgery.

## Abstract

**Background:** North-eastern region of India continue to suffer from limited resources, added upon by Mongoloid racial similarity and poor cataract surgery rate has contributed to the increase incidence of advanced cataract like phacomorphic glaucoma.

**Objective:** To evaluate the visual prognosis and related complications of phacomorphic glaucoma cases by manual small incision cataract surgeries in North-eastern region of India.

**Materials and methods:** This retrospective case series study includes 65 phacomorphic glaucoma cases diagnosed between June 2009 to December 2011 in Jawaharlal Nehru Institute of Medical Sciences, Manipur. Preoperative routine evaluation includes slit-lamp biomicroscopy, measurement of IOP, gonioscopy of fellow eye, axial length measurement, AC depth and lens thickness by A-scan. Manual small incision cataract surgery with intraocular lens implantation was performed in all the 65 eyes. Complete ophthalmic examination was done at each follow-up visit.

**Results:** The mean preoperative IOP was 35.14 (±6.35) mm Hg and IOP at the 3rd month follow-up was 18.65 (±1.3) mm Hg with a statistically significant lowering of IOP (p < 0.0001) at the last follow-up. Intraoperative complications were minimal. Corneal edema, fibrinous exudates in AC was seen in few cases. Postoperative best corrected visual acuity was 6/6-6/12 in 46 eyes, 6/18-6/36 in 12 eyes, 6/60-3/60 in 4 eyes and less than 3/60 in three eyes. Antiglaucoma medication was discontinued immediately after each surgery.

**Conclusion:** Manual small incision cataract surgery being inexpensive play a key role in management of poor outreach program marker like phacomorphic glaucoma in effectively controlling the IOP and achieving good visual acuity with minimal complications.

**How to cite this article:** Rajkumari V, Kaminibabu KS, Bhabanisana RD, Victor R. Manual Small Incision Cataract Surgery in Phacomorphic Glaucoma: Surgical Technique and Outcome in North-eastern India. J Current Glau Prac 2013; 7(2):43-48.

## INTRODUCTION

The estimated number of people visually impaired in the world currently is 285 million in which 39 million peoples are blind and 246 million people have low vision (<6/18 ≥ 3/60). Age 50 years and older constitute the 65% of people visually impaired and 82% of all blind population.^1^ Cataract alone account for 51% causes of global blindness.^1^ Cataract has been documented to be the most common cause of bilateral blindness in India.^2-5^

According to a report in 2001-03, the North-eastern Indian states had combined bilateral blindness of 82.49% in 50+ population.^6^ The statewise prevalence of blindness in the 50+ population was Arunachal Pradesh: 17.56%, Assam: 23.48%, Manipur: 10.66%, Meghalaya: 5.72%, Mizoram: 6.03%, Nagaland: 8.09%, Sikkim: 4.99% and Tripura: 5.96%. Extrapolating the results of the survey to the general population, it was estimated that the 1.38% of the North-eastern population was blind (1.1% blind, overall generalized Indian population).^6^ Even though the most common cause in this population is cataract, the number of cataract surgeries in these regions is far from the target.

In 2003 to 04, the cataract surgery rate (CSR) for Assam was 837, Manipur 220, Arunachal Pradesh 599, Meghalaya 510 and Sikkim 443 respectively.^7^ The recent CSR shown in VISION 2020 12th 5-year plan was disappointing with Assam, Manipur, Arunachal Pradesh, Meghalaya, Sikkim showing CSR < 200/lakh population but Tripura and Mizoram were in a fairly better position with CSR between 200 to 500/lakh population.^8^ The reason being multifactorial namely limited number of surgeons, skilled manpower and limited facilities restricted to some tertiary level hospitals. Poor CSR can be one of the factors for increased incidence of advanced cataract like phacomorphic glaucoma which imposes extra challenge to the resource available in the region.

Phacomorphic glaucoma is highly prevalent in developing countries as has been documented earlier.^9-11^ In India, a study has shown a prevalence of 3.91% phacomorphic glaucoma cases per operated cases for cataract surgery.^12^ Phacomorphic glaucoma is usually caused by lens swelling in the eye due to intumescent cataract. When the lens swells, angle-closure glaucoma with pupillary block occurs in the acute phase, in the late phase, it can occur without pupillary block as a result of forward movement of the peripheral iris.^13^

Management of phacomorphic glaucoma cases includes initial lowering of IOP followed by definitive treatment of removal of lens by cataract surgeries. The potential intraoperative complications like expulsive hemorrhage, weakened zonules tear and unpredictable visual outcome makes management of such cases a challenging procedure.^14,15^ The initial lowering of IOP is commonly done with medical treatment with combinations of topical antiglaucoma medications, oral acetazolamide, intravenous mannitol but in 37.5% cases medical treatment has failed to show signs of improvement.^10^ Equally effective is the laser pretreatment advocated by many surgeons and rarely a procedural syringing.^16-18^

Anterior capsule visualization by trypan blue staining (also indocyanine green 0.5%) has turned a risky procedure of creating a continuous curvilinear capsulorhexis in manual small incision cataract surgery to a relatively safe and economical procedure though there has been some reported association of postoperative IOP elevation and persistent AC inflammation.^19,20^

This study was carried out to evaluate the visual prognosis and related complications of phacomorphic glaucoma cases by manual small incision cataract surgeries in North-eastern region of India where relatively no data is available. We have attempted to quantify the actual volume of phacomorphic glaucoma cases in our surgeries though shortcoming is inevitable while projecting it to the general population for many reasons one being nonuniformity of study population and other being inherent limitation of retrospective studies like incomplete records and inaccurate documentations despite all possible efforts.

## MATERIALS AND METHODS

This retrospective study included all patients with phacomorphic glaucoma from June 2009 till December 2011. A total of 65 eyes were detected to be phacomorphic glaucoma at Jawaharlal Nehru Hospital of Jawaharlal Nehru Institute of Medical Sciences, Imphal, Manipur. Clinical presentation included acute pain on previously painless progressive diminution of vision and redness of eye. Examination revealed circumcorneal congestion, corneal edema, shallow anterior chamber, an intumescent cataractous lens pushing forward the iris and proportionately raising the intraocular pressure (IOP) above 21 mm Hg.^11,15,21^

The preoperative assessment includes pupillary reaction, slit-lamp biomicroscopy, measurement of IOP (Schiotz tonometry), axial length measurement, anterior chamber depth and lens thickness by A scan (Echorule 2, Biomedix Optotechnik and Devices, Bengaluru, India), AB scan to examine the vitreous and retina.^15^ A gonioscopic examination was often not feasible in the presence of corneal edema and was not use for diagnose but to rule out primary angle closure change in fellow as an exclusion criteria.^22^ Initial management of IOP > 35 mm Hg was done medically and with 20% mannitol in a case of persistently high IOP > 45 mm Hg to lower IOP < 25 mm Hg. Written informed consent was taken from all the patients prior to the surgery. The surgeries were performed under retrobulbar anesthesia with facial block.

The surgical steps of MSICS have previously been described.^23,24^

Following a superior fornix based conjunctival flap, a frown scleral incision of 6.5 mm partial thickness was made 2 mm away from the limbus and scleral tunnel was extended 1 mm into clear cornea. Paracentesis was made at the 10 o'clock position. The anterior capsule was stained with trypan blue 0.6% for better visualization. The anterior chamber was filled with viscoelastic substance and continuous curvilinear capsulorhexis was made on the anterior lens capsule by capsulorhexis forcep. A sinskey hook was used to hook out one pole of the nucleus out of the bag and the rest of the nucleus was rotated out in the anterior chamber. The nucleus was extracted out of the eye with irrigating vectis. After aspirating the remaining cortex, a 6 mm optic polymethyl methacrylate posterior chamber intraocular lens was implanted in the bag. The viscoelastic was aspirated and the side port hydrated with balance salt solution. The conjunctival flap was apposed and subconjunctival injection of gentamicin and dexamethasone was given.

Postoperatively patients were treated with topical antibiotic steroidal drops flurbiprofen. All glaucoma medications were discontinued immediately after the cataract extraction. Complete ophthalmic examination was done at 1st postoperative day and each follow-up visits of 1st week, 1st month, 3rd month and 6th month and included visual acuity test, IOP measurement, slit-lamp examination and funduscopy examination.

## STATISTICS

SPSS version 16.0 was used for data analysis. The p-value of <0.05 was considered as significant.

## RESULTS

Out of 1,350 operated cases during the period of 2 years and 6 months for cataract cases, 65 were phacomorphic glaucoma cases (4.81% per operated cases for cataract surgeries). The number of female patients was 37 (56.9%) and that of male was 28 (43.1%) with female to male ratio of 1.3:1. The mean age of presentation for male was 66.54 (±8.2) years and that of female was 63.37 (±7.9). The composite detail of total number of patients, age and sex, duration of symptom and preoperative IOP of the study population has been summarized in Table 1.

### Duration of Symptoms and Preoperative IOPs

Of the 65 cases, maximum number of the phacomorphic glaucoma cases presented at the first week. A total of 53 (81.5%) cases in 0 to 10 days, 10 (15.4%) in 11 to 20 days and remaining 2 (3.1%) cases in 21 to 30 days. The preoperative IOP was between 25 and 35 mm Hg in 17 (26.2%) males and 26 (40%) female patients, 36 and 45 mm Hg in 9 (13.8%) male and 11 (16.9%) female patients and 46 and 55 mm Hg in only 2 (3.1%) male patients. Overall the preoperative mean IOP was 35.1 (±6.4) mm Hg.

**Table 1:** **Table 1:** Number of cases, age and sex, duration of symptom and preoperative IOP

*Sex (total no. of cases)*		*Age (years)*		*No. of cases (%)*		*Duration of symptom*				*Preoperative IOP*			
						*(Days)*		*Patient (%)*		*(mm Hg)*		*Patient (%)*	
Male^28^		50-59		02 (3.1)		0-10		21 (32.3)		25-35		17 (26.2)	
		60-69		20 (30.7)		11-20		7 (10.8)		36-45		09 (13.8)	
		>70		06 (9.2)		21-30		–		46-55		02 (3.1)	
Female^37^		50-59		05 (7.7)		0-10		32 (49.2)		25-35		26 (40)	
		60-69		23 (35.4)		11-20		3 (4.6)		36-45		11 (16.9)	
		>70		09 (13.8)		21-30		2 (3.1)		46-55		–	

### Presenting Visual Acuity and Associated Systemic Illness

Out of the 65 patients, accurate projection of rays was seen in 56 (86.15%) patients and nine (13.85%) patients had inaccurate projection of light. Of the total, 8 (12.3%) were isolated diabetic patients, 5 (7.7%) were both diabetic and hypertensive and 11 (16.9%) were isolated hypertensive patients. A male patient with HIV seropositive was on ART. The preoperative fellow (nonattacked) eye status was summarized along with the presenting visual acuity (PVA) and associated medical illness in Table 2.

### Axial Length and Anterior Chamber Depth

The mean axial length was 22.9 mm (SD 0.7) for female and 23.1 mm (SD 1.0) for male and the mean anterior chamber depth was 1.9 (SD 0.2) for phacomorphic eye taken as average of three consecutive reading. Since more than 95% of patients presented for first time, measurement of lens thickness was not consider significant as it can-not rule out the possibility of thicker lens prior to phacomorphic attack.^25^

**Table 2:** **Table 2:** PVA, fellow nonattack eye status and associated medical illness

*Presenting visual acuity*		*Number (%)*	
Accurate projection of rays		56 (86.2)	
Inaccurate projection of rays		09 (13.8)	
*Fellow (nonattack) eye status*			
Immature cataract		46 (70.8)	
Mature cataract		11 (16.9)	
Pseudophakia		07 (10.8)	
Clear lens		01 (1.5)	
*Associated systemic medical conditions*			
Diabetic		08 (12.3)	
Hypertension		11 (16.9)	
Both diabetic and hypertensive		05 (7.7)	
HIV seropositive (on ART)		01 (1.5)	
Tuberculosis (ATT completed)		01 (1.5)	
No systemic illness		39 (60)	

*Note*: HIV: Human immunodeficiency virus; ART: Antiretroviral treatment; ATT: Antitubercular treatment

**Table 3:** **Table 3:** Intraoperative difficulties and complications along with postoperative complications

*Intraoperative difficulties*		*No. of cases*	
Wound insufficiency		3 (4.6%)	
Incomplete cortical clean up		2 (3.1%)	
Capsulorhexis extension		5 (7.7%)	
Hard cataract and prolapsing nucleus difficulties		3 (4.6%)	
Pupillary constriction		4 (6.2%)	
Iris prolapse		2 (3.1%)	
Posterior capsular tear		2 (3.1%)	
*Postoperative complications*			
Striate keratopathy		7 (10.8%)	
Posterior capsular opacification		3 (4.6%)	
Cystoid macular edema		2 (3.1%)	
Decentralization of lens		1 (1.5%)	

### Intraoperative Difficulties and Complications

Intraoperative difficulties encountered during surgery are wound insufficiency 3 (4.6%) cases, corneal edema and incomplete cortical clean up 2 (3.1%) cases, capsulorhexis extension to the periphery 5 (7.7%) cases, hard cataract and difficulty in prolapsing the nucleus 3 (4.6%) cases, pupillary constriction 4 (6.2%) cases and iris prolapsed 2 (3.1%) cases. Intraoperative complications were minimal. Premature entry to the anterior chamber in 3 (4.6%) cases and posterior capsular rent in 2 (3.1%) cases were the complications encountered during the surgery. The postoperative complications include striate keratopathy 7 (10.8%) cases, posterior capsular opacification 3 (4.6%), cystoids macular edema 2 (3.1%) and decentralization of lens 1 (1.5%). Intraoperative difficulties and complications along with postoperative complications have been summarized in Table 3.

The final postoperative BCVA was 6/6-6/12 in 46 (70.77%) eyes, 6/18-6/36 in 12 (18.46%) eyes, 6/60-3/60 in 4 (6.15%) eyes and finger counting (1/60)/hand movement or projection of light in 3 (4.62%) eyes. Comparison between the postoperative BCVA, duration of symptom and preoperative IOP has been summarized in Table 4. The mean IOP at 3rd month follow-up was 18.65 (±1.3) mm Hg. Three out of 65 defaulted after 3rd month follow-up. There was a statistically significant difference between IOP at presentation and last follow-up visit (p < 0.0001, paired t-test).

**Table 4:** **Table 4:** Comparison between the postoperative BCVA, duration of symptom and preoperative IOP

*Postoperative BCVA 90th days PO*		*Preoperative IOP*						*Duration of symptoms*					
		*25-35 (mm Hg)*		*36-45 (mm Hg)*		*46-55 (mm Hg)*		*0-10 (days)*		*11-20 (days)*		*21-30 (days)*	
6/6-6/12		35		10		01		44		02		–	
6/18-6/36		06		06		–		08		04		–	
6/60-3/60		01		02		01		–		03		01	
FC/HM/LP		01		02		–		01		01		01	

*Note*: FC: Finger counting; HM: Hand movement; LP: Light perception

## DISCUSSION

Extracapsular cataract extraction was not perform because of the risk associated with the large incision (12-14 mm) and sight threatening complications due to high IOP like expulsive hemorrhage.^26,27^

Well control studies have clearly shown the advantage of manual small incision cataract surgery over extracapsular cataract extraction in cataract surgery in terms of visual outcome and postoperative recovery which is crucial in such cases.^28^

Phacoemulsification (clear corneal temporal) has advantage over both ECCE and MSICS in reduced conjunctival scaring for subsequent filtering surgeries if at all required.^29^

The greater surgical risk imposed by phacomorphic angle closure especially in the setting of any residual corneal edema, dense and hard nuclei and weaken zonules added upon by its endothelial damaging ability of ultrasound energy because of the close proximity of the phaco tip during nucleus emulsification make the phacoemulsification procedure unfavoring in our setting.^30^

Manual small incision cataract surgery with trypan blue staining of the anterior lens capsule has a stable anterior chamber because of its shelving scleral wound besides being economical. Venkatesh et al and Ramakrishan R et al have shown the efficacy of MSICS in management of phacomorphic glaucoma cases in terms of visual outcome and safety.^23,24^

The incidence of phacomorphic glaucoma per operated case for cataract was higher than the previous study by Jain IS et al by 0.9% which could be due to the racial similarity with the mongoloid wereas many studies has reported the relative risk of increase incidence of glaucoma cases due to smaller axial length and anterior chamber depth.^12,30,31^ Perhaps more important cause could be the increase backlog of poor CSR.^7,8^

Lack of biometric data relating axial length distribution among local population has limited our study more so with the possible variation within the same ethnicity and also within a population based on differences in age, gender, refraction error and corneal curvatures. Axial length has been a significant predictor for various forms of glaucoma but such significance is not established for phacomorphic glaucoma which can be seen in narrow angles and open angles both.^16,25^ Lee et al found that eye with axial length shorter than the population mean were 4.3 times as likely to develop phacomorphic glaucoma compared with eyes with longer than average axial length which they have taken as 23.7 mm for patient of Chinese ethnicity.

For unknown reason, our study have found that female 37 (56.9%) predominate male 28 (43.1%); a similar finding by Ramakrishanan R et al where they have found female to male ratio of 2.41:1 in there study.^24^

The final visual acuity was better in those groups which had shorter duration between the onset of symptoms and surgery (linear regression, p = 0.000, r^2^ = 0.5). In the third month postoperative follow-up, 83.02% achieving good visual acuity in presentation <10 days of onset, 3.07% of those presented after 10 days could achieve BCVA 6/12 or better. Of the total 7 (10.77%) patients had poor visual recovery of <3/60 with glaucomatous optic changes have been found in one eye, cystoid changes in macula in two eyes, pre-existing diabetic retinopathy in one eye and posterior capsular opacification in three eyes which subsequently under went laser treatment.

The IOP on the final 6th month postoperative follow-up did not show much variation from the IOP of 3rd month follow-up, ranges from 17.3 to 20.6 mm Hg with the mean IOP of 18.8 (±1.6) mm Hg. In all the cases the IOP was controlled without the need of long-term antiglaucoma medications.

## CONCLUSION

Manual small incision cataract surgery is effective in controlling IOP and achieving good visual acuity with minimal complications in the management of phacomorphic glaucoma. MSICS being safe and inexpensive play a key role in checking the poor outreach program marker like phacomorphic glaucoma and allow better quality of life to this section of population which need early diagnosis and interventions.
